# Calcium Wave Propagation in Networks of Endothelial Cells: Model-based Theoretical and Experimental Study

**DOI:** 10.1371/journal.pcbi.1002847

**Published:** 2012-12-27

**Authors:** Juexuan Long, Michael Junkin, Pak Kin Wong, James Hoying, Pierre Deymier

**Affiliations:** 1 Material Science and Engineering, University of Arizona, Tucson, Arizona, United States of America; 2 Aerospace and Mechanical Engineering, University of Arizona, Tucson, Arizona, United States of America; 3 Biomedical Engineering and Bio5 Institute, University of Arizona, Tucson, Arizona, United States of America; 4 Cardiovascular Innovation Institute, University of Louisville and Jewish Hospital, Louisville, Kentucky, United States of America; Northwestern University, United States of America

## Abstract

In this paper, we present a combined theoretical and experimental study of the propagation of calcium signals in multicellular structures composed of human endothelial cells. We consider multicellular structures composed of a single chain of cells as well as a chain of cells with a side branch, namely a “T” structure. In the experiments, we investigate the result of applying mechano-stimulation to induce signaling in the form of calcium waves along the chain and the effect of single and dual stimulation of the multicellular structure. The experimental results provide evidence of an effect of architecture on the propagation of calcium waves. Simulations based on a model of calcium-induced calcium release and cell-to-cell diffusion through gap junctions shows that the propagation of calcium waves is dependent upon the competition between intracellular calcium regulation and architecture-dependent intercellular diffusion.

## Author Summary

Calcium wave signal has been found in a wide variety of cell types. Over the last years, a large number of calcium experiments have shown that calcium signal is not only an intracellular regulator but is also able to be transmitted to surrounding cells as intercellular signal. This paper focuses on the development of an approach with complementary integration of theoretical and experimental methods for studying the multi-level interactions in multicellular architectures and their effect on collective cell dynamic behavior. We describe new types of higher-order (across structure) behaviors arising from lower-order (within cells) phenomena, and make predictions concerning the mechanisms underlying the dynamics of multicellular biological systems. The theoretical approach describes numerically the dynamics of non-linear behavior of calcium-based signaling in model networks of cells. Microengineered, geometrically constrained networks of human umbilical vein endothelial cells (HUVEC) serve as platforms to arbitrate the theoretical predictions in terms of the effect of network topology on the spatiotemporal characteristics of emerging calcium signals.

## Introduction

Multi-level organization and dynamics is a hallmark of most biological systems. This is particularly true in tissues in which single cells are organized into multicellular structures, which are further assembled into complex tissue and organs. For example, endothelial cells are assembled into multicellular tubes (i.e. vessels) which are connected to each other to form a branched vascular tree system. Molecular signals are initiated and/or processed at the endothelial cell level yet influence overall tree behavior and vice-versa [1]. Central to the proper behavior in these biological systems is cross-level interdependence. To date, limited studies of signaling in multicellular networks have demonstrated that the architecture of multi-cellular systems have a significant impact on the behavior of individual cells as well as their emerging collective behavior.

Over the past decade, questions concerning the system behavior of cellular structures have received increasing attention. For instance, there is strong evidence that the branching architecture of the mammary gland is a major regulator of normal epithelial cell signaling and function [2], [3]. Normal organ architecture can suppress tumor formation and prevent malignant phenotypes even in grossly abnormal cells [4]. Tissue engineering in its attempt to construct functional tissues faces the challenge of arranging cells (e.g. scaffolding via decellularization of allograph tissue) in a three-dimensional configuration with architecture analogous to the native tissue to support proper spatial and temporal molecular signaling necessary to sustain appropriate development and function [5]. Also, downstream and upstream signal conduction between endothelial cells along the walls of vessels plays an important role in microcirculatory function, vascular network remodeling, vasculogenesis, and neovascularization [6].

A particularly relevant aspect to tissue engineering is the emerging behavior of a multicellular architecture in which cell-level functions, such as intracellular communication, integrate with multicellular architectures through local cell-to-cell interactions. Central to this problem is that cellular networks inherently combine dynamical and structural complexity. Early progress on modeling coupled dynamical systems was limited to space-independent coupling or regular network topologies. Further progress to circumvent the difficulty of modeling associated with the combined complexity of the dynamics and of the architecture was achieved by taking a complementary approach where the dynamics of the network nodes is set aside and the emphasis is placed on the complexity of the network architecture [7]. Accordingly, linear solutions of calcium reaction/diffusion models of multicellular architectures composed of networks of chains of cells with grafted side branches have shown that calcium wave propagation differs in ordered or disordered architectures [8], [9]. Similar effects have also been encountered in chains of endothelial cells with non-linear intracellular calcium dynamics [10].

To evaluate the effects of multilevel architectures on biological signal behavior, we modeled calcium-signal propagation in networks of endothelial cells experimentally and computationally. The vasculature is an ideal system for evaluating multi-scale behavior given the relatively simple but multi-ordered organization of the cells and tissues. Here, the behavior of a calcium wave moving along branched chains of endothelial cells was simulated using experimentally observed parameters in the computation. While there are numerous stimuli that can initiate calcium waves in endothelial cells, we utilized the mechanical stimulation of a single endothelial cell as the wave initiator to minimize confounding issues related to multiple upstream and downstream effects intrinsic to diffusible (i.e. pharmacological) signals. Furthermore, mechanical forces play important roles in endothelial function in vivo [11]. The theoretical aspect leverages progress in modeling of the dynamics of complex networks and in microengineering of multicellular structures to generate new knowledge concerning multicellular architectures.

## Methods

### Experimental Method

Our study is based on networks of human umbilical vein endothelial cells (HUVEC) (ATCC CRL-1730) in which intercellular calcium wave propagation is primarily dominated by gap junction [12].

#### Surface patterning

**Figure 1:**
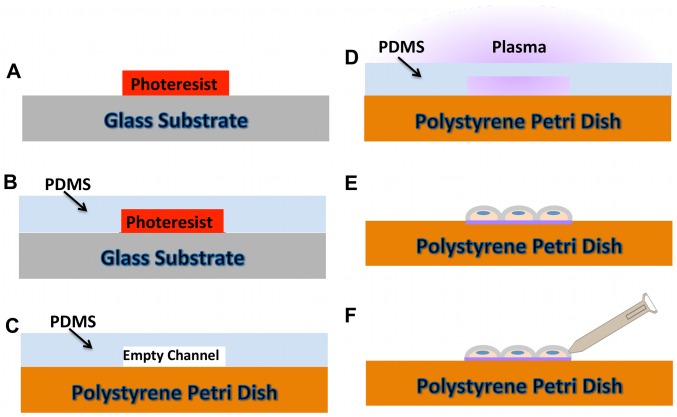
Plasma lithography for cell patterning. (A) Photolithography is used to form a template of the desired multicellular network. (B) PDMS is poured over the photoresist pattern to create an initial plasma shielding model. (C) PDMS mold is transformed onto a Petri Dish (polystyrene). (D) The plasma surface treatment is used to produce cell-sensitive chemical pattern on the area of PDMS mold. (E) Cell seeding. (F) Cell stimulation.

The experimental investigation of multicellular calcium ion propagation relies on organizing multiple cells into specific configurations via a surface patterning technique (Figure 1), which guided cellular attachment. Selective plasma functionalization of surfaces was employed to obtain the surface-patterning component of the system and consists of forming patterns of surface groups on biocompatible polymers [13], [14], [15], [16], [17]. The patterning is achieved via controlling contact of reactive plasma to a surface in order to spatially change surface chemistry. This process uses photolithography to first produce microscale patterns, which are designed, based on where cell attachment is desired. Patterning molds are then created by replica molding of polydimethylsiloxane (PDMS) onto the microscale resist structures. This produces a PDMS shape having 3D microscale topography. This 3D structure is then placed on the culture surface with a weight to ensure conformal contact and proper formation of channels between itself and the surface. The channels formed between the mold and culture surface ultimately determine where new functional groups are introduced when placed in a plasma chamber. In the case of the polystyrene substrates used in this work, select areas of native polystyrene come in contact with air plasma. This creates patterns alternating between a surface similar to tissue culture treated polystyrene, and native polystyrene, which, as it has not been modified by the plasma, is cell repulsive. For the current experiments patterns were produced on the bottom of polystyrene Petri dishes (VWR 25384-090) using PDMS (Dow, Sylgard 184) 3D shapes for shielding. The molds used to produce these 3D shapes were created from AZ3312 resist (AZ Electronic Materials) spun onto glass slides. The patterns were designed to be approximately one cell width wide and so had channel widths of 20–30 µm. The plasma treatment took place with air plasma for ten minutes at 150 Pa using a Harrick plasma chamber (model PDC-001) followed by ten minutes of UV sterilization before seeding with cells. Cells were detached with 0.25% trypsin-EDTA (Invitrogen), seeded onto patterned Petri dishes and incubated with standard culture medium for one day before stimulation. Standard culture medium consisted of F-12K Medium (ATCC) supplemented with 20% screened FBS (Gemini Bio-Products), 0.1 mg/ml heparin (Sigma-Aldrich), 0.035 mg/ml endothelial cell growth supplement (Sigma-Aldrich), and 0.1% gentamycin (GIBCO).

#### Cell stimulation

**Figure 2:**
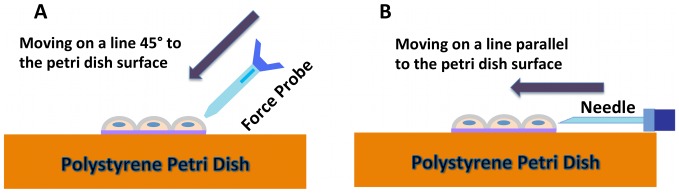
Schematic of cell stimulation. (A) Force probe stimulation. The direction of stimulation is from above the cells, moving 45° to the tissue culture Petri dish surface. (B) Needle stimulation. The direction of movement is from the side, parallel to the tissue culture Petri dish surface.

In the current study, mechanical stimulation is used to trigger calcium release from internal stores in single cells (Figure 1(F)). We term the probed cell, “the stimulated cell”, and mechanical stimulation was achieved in either of two ways (Figure 2). The first was by probing with a force sensor (FemtoTools Instruments, FT-S540), which provided a measurement of force applied to cells during stimulation. It was used to test the influence of probing force on calcium signal propagation (Figure S1). The second probing method used a 30-gage syringe needle. Since the physical size of stainless steel needle is smaller than force probe, it was used in most of our experiments including the experiments shown later to get better visualization of cells during probing compared to probing with the force probe. The operation of the force probe required contact to a cell from above and would obscure visualization of cells adjacent to the stimulated cell. Stimulation with a syringe needle allowed the needle to be brought into contact with the stimulated cell from the side of the cell and consequently did not obscure imaging of calcium in adjacent cells. Both of these probes were attached to a custom three-axis micromanipulator for placement and stimulation. The probe would be brought near to a pattern of cells, and imaging would be initiated for baseline gathering. Fluorescence images would then continue to be captured during and after stimulation.

#### Imaging

Fluro-3AM was loaded into cells as a Ca^2+^ sensitive dye to visualize the small messenger signaling. Once loaded, esterases cleave the dye so that it cannot leave the cell and will fluoresce in the presence of Ca^2+^. A Nikon TE2000-U inverted phase contrast and fluorescence microscope equipped with a Cooke SensiCam was used to capture the images. Cells were first plated on patterned surfaces as described above and maintained in standard culture medium. Dye loading was initiated by adding 5 or 10 µl of a 1 mg/ml solution of Fluo-3AM (Invitrogen) dissolved in dimethyl sulfoxide (DMSO), (Fisher) and 5 or 10 µl of a 10 mg/ml solution of Pluronic F-127 (Invitrogen) dissolved in deionized water, to the three milliliters of standard culture medium contained in the patterned dish. The cells were then incubated for 15 minutes at 37°C before being gently rinsed with standard culture medium. New culture medium was added and the cells were incubated for ten minutes at 37°C. Medium was then exchanged and cells were incubated for another ten minutes at 37°C. Finally cells were rinsed with HBSS without calcium or magnesium (Fisher) and the same HBSS was added for imaging, which took place on a microscope stage heated to 37°C. Prior to stimulation, images were captured in phase contrast to visualize cell outlines and during stimulation, illumination was changed to fluorescence with a filter cube providing excitation at 460–500 nm and emission at 510–560 nm. Images were captured every 1.2 seconds and exported for later analysis. For analysis, cells were manually outlined in image sequences and analyzed using Image J, which exported integrated fluorescence versus time values for later analysis. Images and probing were obtained within ∼25 minutes of the final buffer rinse and cells were imaged at passage six or lower.

Whole cell fluorescence intensities as a function of time are obtained by integrating the intensity of every pixel over the area of each cell corrected for the integrated fluorescence of a background area and normalized by the initial intensity. In absence of a one-to-one correspondence between normalized fluorescence intensity and the level of cytosolic calcium concentration, and in light of the cell-to-cell variability in the intensity of the fluorescence, the magnitude of fluorescence is not always taken as a measure of the response of a cell. Irrespective of the magnitude of the fluorescence, a cell is considered to have responded when it exhibits a temporal fluorescence response constituted of an initial fast rising stage followed by a slower decline in fluorescence (see section “Chain Subjected to Single Stimulation: Experiments” for details). The response time of a cell is then determined as the time at which fluorescence reaches its maximum positive rate of change. For weak temporal responses that exhibit significant noise levels, the noise to signal ratio is improved by calculating the running average of the rate of change of the fluorescence. The time average is performed on four time intervals. Response times determined from the running average will therefore be associated with larger uncertainties of 1.2 s.

### Computational Model and Method

Since we are interested in the behavior of networks of endothelial cells composed of one-dimensional chains of cells and networks of chains of cells, a reaction/diffusion model is developed to gain insight into the architecture-dependence of calcium wave propagation. For the sake of simplicity, we only consider the dynamic of intracellular calcium and assume the intercellular Ca^2+^ is transported between cells by diffusion through gap junctions.

#### Numerical models

he model is based on the one-dimensional time-dependent reaction/diffusion equation:
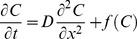
(1)where *C* is the cytosolic calcium concentration, *D* is a coefficient representing diffusion between cells through gap junctions, and *f* is the rate of change of intracellular calcium concentration. *x* and *t* are the position and time variables.

**Figure 3:**
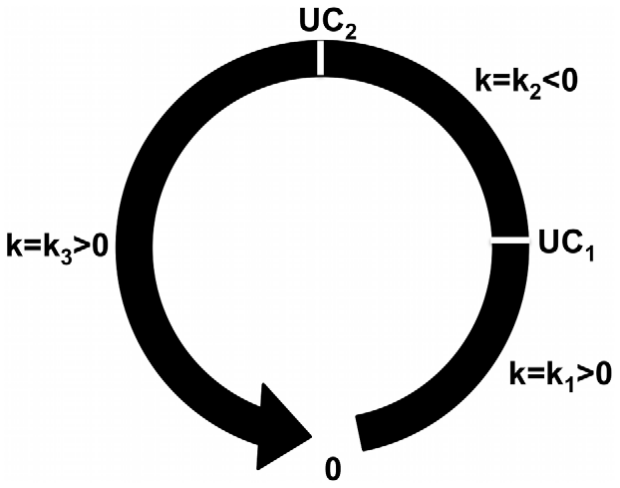
Schematic representation of the non-linear intracellular calcium reaction dynamics as a loop in calcium concentration space. UC_1_ and UC_2_ are lower and upper thresholds of intracellular calcium concentration, respectively, which determine the value of calcium release/intake rate constant, *k*.

Intracellular calcium dynamics results from the response of Ca^2+^ stores, primarily the endoplasmic reticulum (ER), to inositol triphosphate (IP_3_) and ryanodine through IP_3_ receptors (IP_3_R) [18] and ryanodine receptors [19]. Both sensitized IP_3_R and RYR, lead to a process whereby calcium can trigger the release of additional calcium from the ER, namely calcium induced calcium release (CICR). Furthermore, since high levels of intracellular calcium are toxic, and cannot be degraded, cells control the intracellular calcium level by buffering, sequestration in specialized compartments, and by expulsion to the extracellular space [20]. Meanwhile, cytosolic calcium concentration is also inhibited by low level of Ca^2+^ [18], [21]. In the basis of these processes, we model the rate of change of intracellular calcium by a simple calcium dependent non-linear reaction function:
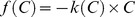
(2)with 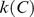 representing a calcium concentration-dependent calcium release/intake rate constant. To model the CICR and calcium buffering processes, we defined two thresholds *UC_1_* and *UC_2_*, which determine the value of the calcium release/intake rate as shown in Figure 3.

*UC_1_* is the threshold below which cytosolic calcium concentration is too low to promote ER release of additional calcium. Therefore, for calcium concentrations below *UC_1_*, the ER absorbs cytosolic calcium and the calcium release/intake rate constant takes on a positive value 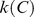 = *k_1_*. The rate of calcium dynamics, *f*, is therefore negative which corresponds to ER calcium intake. Calcium is released from ER to the cytosol, when the cytosolic calcium concentration is within the range *UC_1_* and *UC_2_*. Within this range, the value of the calcium release/intake rate constant, 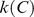 = *k_2_* is negative and *f* is positive. This corresponds to release of calcium by the ER and the total concentration of cytosolic calcium increases. If the intracellular calcium level exceeds the threshold *UC_2_*, then the cytosolic calcium is again absorbed into ER or extruded to extracellular space. Within this range, the value of the calcium rate constant, 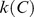 = *k_3_* is positive and the rate of the cytosolic calcium dynamics is negative. Because cells exhibit a refractory stage of approximately 30 s after a complete cycle of calcium release followed by intake [22], in our model once the calcium rate constant reaches the value k_3_, the cell will retain that value indefinitely.

In the case of a spatially discrete representation of multicellular chain and considering diffusion between nearest neighbor only, equation (1) is discretized in space and time using finite differences and takes the form:
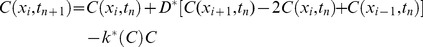
(3)


**Figure 4:**
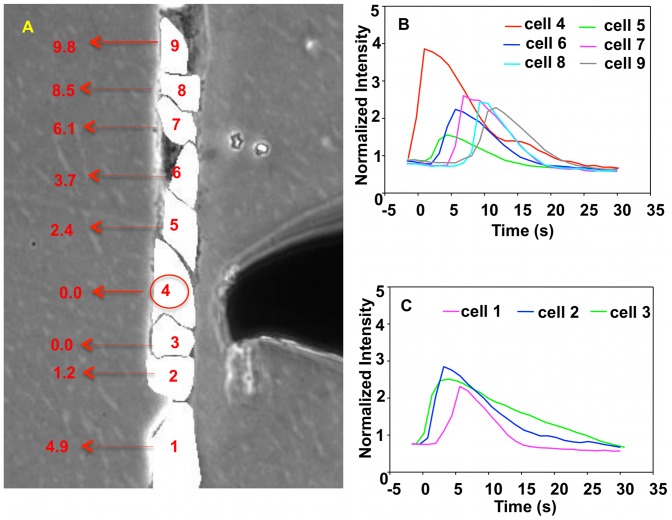
Experiment image and normalized intensity of cells in single fine line subjected to single stimulus. (A) Image of a finite single fine line of cells. Cells are labeled 1 through 9. The stimulating probe is clearly visible on the right of the stimulated cell (cell 4 labeled with a red circle). The time (in sec) at which the normalized fluorescence reaches its maximum positive rate of change is indicated for every cell. The uncertainty for each one of these times is 0.6 s. The origin of time is the time at which fluorescence in the stimulated cell attains its maximum rate of change. (B) and (C) show the normalized intensity of fluorescence of cells 4 through 9 and cells 3 through 1, respectively, as functions of time. The vertical axis is the dimensionless normalized intensity of fluorescence and the horizontal axis is the time in seconds in intervals of 1.2 s between recordings.

The term on the left-hand side is the concentration of Ca^2+^ in cell “*i*” at time *t_n+1_*, and all the terms on the right-hand side are at a time *t_n_*. The time step is denoted 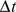. The concentration for the next time increment, *n+1*, can be calculated from values of concentration at the previous time increment, *n*. 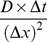 is defined as a dimensionless diffusion coefficient denoted “
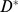
”. 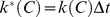 represents a dimensionless rate constant. For linear chains of endothelial cells, we assume that the calcium diffusion coefficient is the same between neighboring cells. However, we will consider spatially dependent diffusion coefficients in the case of models of more complex architectures such as “T” structures. This more complex model is detailed in the Appendix S1. Calcium signals described by our model do not exhibit attenuation since we observed variations in the fluorescence intensity in individual experiments. Nevertheless, our model is able to capture the major features of intercellular calcium propagation described in the paper (see Figure 4).

## Results

We investigate the behavior of several types of multicellular structures, namely single chains of endothelial cells and “T” structures subjected to either single or simultaneous double mechano-stimulation at different locations in the structures.

### Chain Subjected to Single Stimulation

In this section we consider the behavior of a finite chain of endothelial cells among which a single cell in the chain is subjected to mechanical stimulation to initiate a calcium impulse, due to the intracellular increase in calcium concentration.

#### Chain subjected to single stimulation: experiments

Figure 4 and Video S1 show the calcium-activated fluorescence of individual cells as a function of time in a finite single fine line of cells with one cell (cell 4) subjected to a single mechano-stimulation. Note that since the fluorescence of the system is recorded every 1.2 s, the response time of every cell in the structure is associated with an uncertainty of 0.6 s.

The observed shape of the calcium pulse is consistent with that previously reported [23]. While we observed some attenuation of the pulse amplitude as a function of distance in several cases as reported by others [22], this did not occur in all experiments (as in Figure 4). The response time of a cell is defined to be the time corresponding to the highest value of the rate of change of fluorescence versus time. The response time of the stimulated cell (cell 4) is set as the origin of time. The response of cells 5 through 9 and 3 through 1 lags behind that of the stimulated cell as an increasing function of the distance from the stimulated cell. Thus two calcium pulses propagate along the cell chain in opposite directions with both pulses originating at cell 4. Figure 4 indicates that calcium pulses can propagate over distances of at least 3 to 5 cells with relatively high intensity beyond the stimulated cell. For 20 different cell chains we examined, calcium pulses propagated from the stimulated cell with an average distance of propagation of 4.7 cells with a standard deviation of 1.1. Furthermore, the degree of force applied (from 3 to 300 µN) to the cell had no significant effect on the distance of propagation of a calcium pulse (Figure S1). This indicates that the propagation of the calcium pulse over a finite distance is not driven by stress/strain induced effects along the chain of cells. Also the normalized fluorescence intensity appears to be independent of the force applied. Based on the experimental data shown in Figure 4, the average time of the propagation of calcium wave from one cell to the next is approximately 1.8 s. Moreover, the average width of a temporal pulse is on the order of 10 to 15 s. This means that a regular pulse may extend spatially over 6 (10/1.8) to 9 (15/1.8) intercellular spacings. The average distance between cells in a single chain is approximately 31.2 µm. Therefore the average speed of a calcium wave is estimated to be on the order of 17 µm/s. It is important to note that the time it takes for the fluorescence signal to propagate from cell to cell is highly variable, differing by a factor of two in some cases. The variation may be due to the variability in cell size, cell state, gap junction distribution in the cell membrane, or other characteristics of the cells.

#### Chain subjected to single stimulation: simulations

The cell chain in the simulation consists of 61 cells aligned in single fine line. To begin the simulation, a pulse is initiated in the middle-most cell of the chain, which is sufficiently far from the edges of the chain to avoid artifacts that could arise from boundary effects. For the ease of comparison with the experimental results, we subsequently label the cells in the chain from 26 through 34 such that the stimulated cell is labeled as cell 4. The initial calcium concentration of all but the stimulated cell is set to zero. The initial calcium concentration of the stimulated cell is *C_0_*, which is greater than *UC_1_*. This initial concentration triggers the CICR process initiating a pulse. The value of the model's parameters was explored until the system exhibited calcium wave propagation with characteristics comparable to those of the experimentally observed wave. In particular, we search for parameters that yield a two-stage calcium pulse: an initial stage showing fast rising calcium concentration followed by a slower decrease. The simulated calcium pulse has a spatial extend of approximately 10 cells comparable to what was observed experimentally.

**Figure 5:**
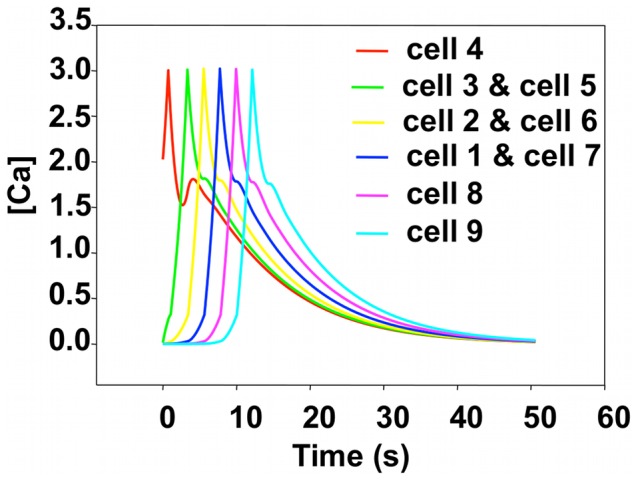
Simulated calcium concentration for cells in a single chain subjected to a single stimulus. We label the stimulated cell 4 (cell 31 in the chain of 61 cells). We report the response of cells on either side of the stimulated cell as cell 5 through cell 9 and cell 1-through cell 3 to facilitate comparison with experimental results. The real time is obtained by scaling the cell-to-cell propagation time of the simulation to that of the experiment (see text for details).

The optimum parameters are reported in Table 1. In the model all rates are dimensionless. The evolution of the calculated calcium concentration in each cell is reported in Figure 5.

**Table 1:** Value of dimensionless parameters used in the reaction-diffusion model of intercellular and intracellular reaction-diffusion dynamics.

Symbol	Definition	Value
C_0_	Initial concentration of calcium in stimulated cell	2.0
UC_1_	Calcium concentration threshold 1	0.3
UC_2_	Calcium concentration threshold 2	3.0
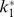	Calcium release/intake rate for C<UC_1_	0.03
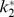	Calcium release/intake rate for UC_1_<C<UC_2_	−0.025
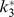	Calcium release/intake rate for C>UC_2_	0.0045
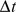	Time interval	0.01
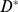	Diffusion coefficient	0.6

From the experimental data, the average time for propagation from one cell to the next one is 1.8 s. In the simulation, the dimensionless time for propagation from one cell position to the next one is 0.87. Thus, we obtain a conversion factor from dimensionless to real time, 
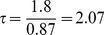
(s). Therefore, the dimensionless integration time step 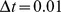 effectively amounts to 0.0207 s. With an average experimental spacing between cells of 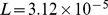
(m) and a dimensionless diffusion coefficient in the simulation of 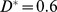, we can calculate the actual value of the diffusion coefficient in our model as 
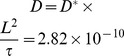
 (m^2^/s). Note that this diffusion coefficient accounts for calcium diffusion between cells through the cytoplasm as well as through gaps junctions. It is in reasonable agreement (in the same order of magnitude) with a free Ca^2+^ diffusion coefficient in a cytosolic extracts from *Xenopus laevis* oocytes of 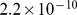 m^2^/s [24].

Propagation is symmetric in the simulation and the response of cells 5 and 3, cells 6 and 2, cells 7 and 1 are the same. There is no variability in the magnitude of the calcium response from cell to cell in the model. The development of a calcium wave that propagates on both sides of the stimulated cell results from the competition between intracellular dynamics and intercellular diffusion. The calcium concentration of the stimulated cell initially exceeding *UC_1_* leads to ER calcium release and therefore a fast rise in the cytosolic calcium in spite of a competition with diffusion that leaks calcium to the neighboring cells. When the concentration of cell 4 exceeds *UC_2_* it declines steadily. Diffusion from cell 4 to cells 3 and 5 increases their respective calcium concentration beyond *UC_1_*, which in turns triggers CICR. This process sustains the propagation of a calcium wave. Note that in the simulation, there is a smaller hump appearing in the tail of each pulse. This behavior, which has no significant effect on the simulation result, is not observed experimentally and is an artifact resulting from the discrete nature of the model. Indeed calcium diffusion is bi-directional and driven by the calcium concentration gradient between neighboring cells. When the calcium concentration level in cells 5 and 3 reaches its highest level, the calcium concentration in cell 4, which is in a declining phase can rise again due to a large calcium diffusion flux originating in cells 5 and 3. This behavior would be more diffuse in a model that would account for the continuous nature of the cell cytoplasm.

### Double Stimulation of Single Chain

We now consider the behavior of a chain of cells subjected to dual mechano-stimulation. The stimulations are applied simultaneously on two cells separated by a short distance. In light of an average distance of propagation of a calcium pulse of approximately 4.7 cells, this distance is chosen so that one could expect possible overlap of the signals emanating from the two stimulated cells in the region separating them.

#### Single chain-dual stimulation: experiments

**Figure 6:**
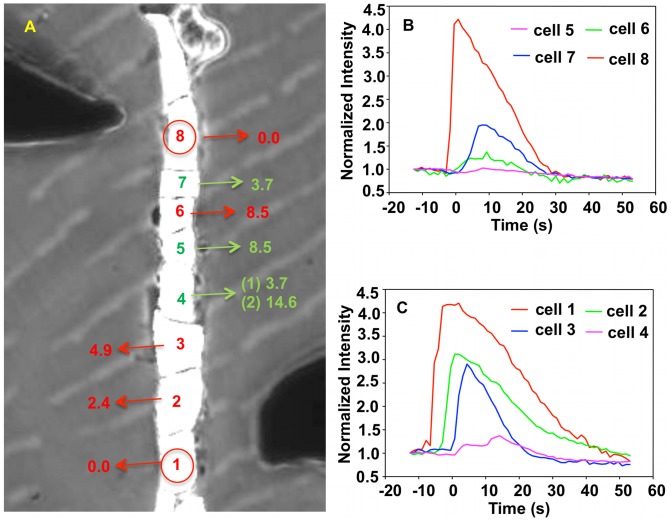
Experiment image and normalized intensity of cells in single fine line subjected to double stimulus. (A) Image of a finite single fine line of cells subjected to dual mechano-stimulation. The stimulating probes are visible at the top-left and bottom-right of the image. The response time of cells labeled in red was calculated from the maximum rate of change of the fluorescence intensity with uncertainties 0.6 s. The response time of cells labeled in green was calculated from a running average of the fluorescence intensity with uncertainties 1.2 s. The first response time for cell 4 (1), represents the time of the first sharp rise in fluorescence versus time. The second response time of cell 4 (2), indicates the time when the fluorescence intensity increases a second time. Cells 4 and 5 represent the region where the calcium pulses are anticipated to meet. (B) and (C) present the normalized intensity of fluorescence of individual cells as a function of time.

Figure 6 and Video S2 show one example of a chain of endothelial cells with dual stimulation. Here, the stimulated cells are separated by 6 cells, which is shorter than two times the average distance of propagation of calcium pulses.

The time evolution of the fluorescence intensity of cells 4 and 5 is significantly different from that of the other cells and of signals observed in the case of a single chain with a single stimulation. As stated in section “Imaging”, the determination of the cell response time from signals with weak intensity is conducted on a running average of the rate of change of the intensity.

In Figure 6 (A), the average time for propagation from one cell to a neighbor of a pulse originating at cell 8 is 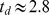 s (the subscript “d” stands for downward propagation), and the average cell-to-cell propagation time of a calcium pulse originating at cell 1 is 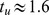 s (the subscript “u” stands for upward propagation). The statistical average times for propagation from one cell to next one based on averaging 17 experimental samples are 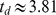 s with standard deviation *STDEV* = 2.32 s and 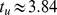 s with standard deviation *STDEV* = 2.11 s. Calcium signals can propagate over an average maximum distance of 5 to 6 cells following a single stimulation event. Because of the difference in upward and downward cell-to-cell propagation time, and considering the possibility of calcium signals that could cross, one would expect to observe a second peak in the response of cell 3 at approximately 17 s, however, this is not the case. The absence of this second peak indicates that once the first pulse has triggered CICR of cell 3, that cell is unable to respond a second time in less than 17 s. This is a characteristic of the refractory behavior of a cell. However, cell 4 appears to exhibit a second peak. This cell is therefore the location where the two pulses meet with a time difference of approximately 10 s. The absence of a second peak in the response of cells 5 and 6 supports this inference. These observations indicate that the two pulses, which propagate toward each other, are unable to pass each other due to the refractory behavior of CICR in endothelial cells. We conducted 26 experiments similar to that reported in Figure 6 and 25 out of 26 of these showed calcium pulses that are not able to cross one another. This result provides strong statistical evidence for the absence of calcium wave crossing in chains of endothelial cells.

#### Single chain-dual stimulation: simulations

**Figure 7:**
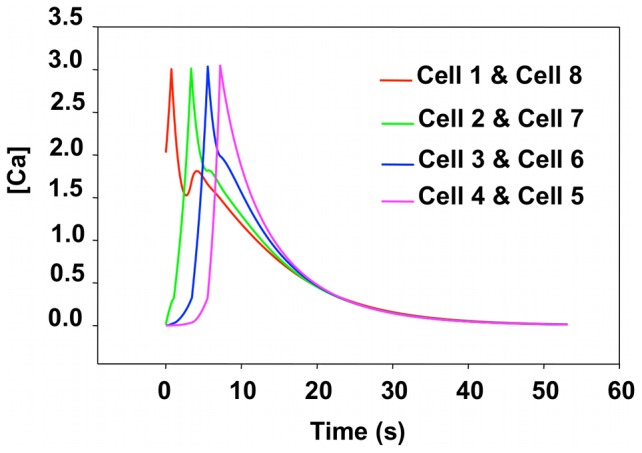
Simulated calcium concentration for cells in a single chain subjected to double stimulus. Cell 1 and cell 8 are the stimulated cells. We report the response of cells between the stimulated cells as cell 2 through cell 7 to facilitate comparison with experimental results. The real time is obtained by scaling the cell-to-cell propagation time of the simulation to that of the experiment (see text for details).

As described in section “Chain Subjected to Single Stimulation: Simulations”, we simulated the behavior of a long chain of cells by modeling a chain with sufficient length to avoid any edge effect during the simulation time when the dual stimulation is applied in its central region, namely cells 1 and 8. All initial calcium concentrations are set to zero except for the stimulated cells, which have an initial calcium concentration exceeding the threshold *UC_1_*. The cells located between the stimulated cells are labeled cells 2 through 7. Due to the symmetry of the model, cells 1 and 8, 2 and 7, 3 and 6, 4 and 5 behave in exactly the same way (See Figure 7).

The response of cell 1 and 8 shows a secondary peak already attributed to the discrete nature of diffusion in our model. If the calcium pulses propagating in the segment between cells 1 to 8 were able to cross one would expect a peak in calcium concentration of cell 1 resulting from the pulse originating at cell 8 at an approximate time of 16 s. The response of cell 1 does not show such a peak. The occurrence of such a peak is not possible since the computational model includes implicitly a refractory stage for CICR. The calcium waves originating from the stimulated cells merge at cells 5 and 4. There, the calcium concentration decays steadily as the rate of calcium dynamics is negative and further response of the cells is prevented. In other words, once the rate of intracellular calcium dynamics, *k_3_*, is reached, a cell cannot respond anymore and the calcium pulses do not cross, as observed in the experimental and computational findings.

### “T” Structure Subjected to Single Stimulation

**Figure 8:**
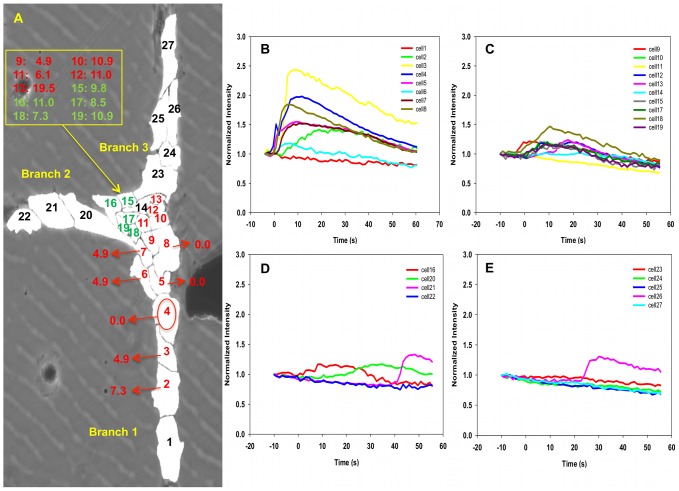
Experiment image 1 and normalized intensity of cells in “T” structure subjected to a single mechano-stimulation. (A) Image of a T structure of cells subjected to single mechano-stimulation. The red circle indicates the location of the stimulated cell. Red labels correspond to cells exhibiting strong fluorescence with response time measured from the rate of change of the fluorescence intensity. Green labels correspond to cells exhibiting weak fluorescence and response time derived from running averages of the rate of change of the fluorescence intensity. Black labels are for cells that show very weak (within the noise level) to no fluorescence. (B–E) shows the normalized intensity of fluorescence of branch 1, cluster area, branch 2 and branch 3, respectively, as functions of time. The vertical axis is the dimensionless normalized intensity of fluorescence and the horizontal axis is the time in seconds in intervals of 1.2 s between recordings.

**Figure 9:**
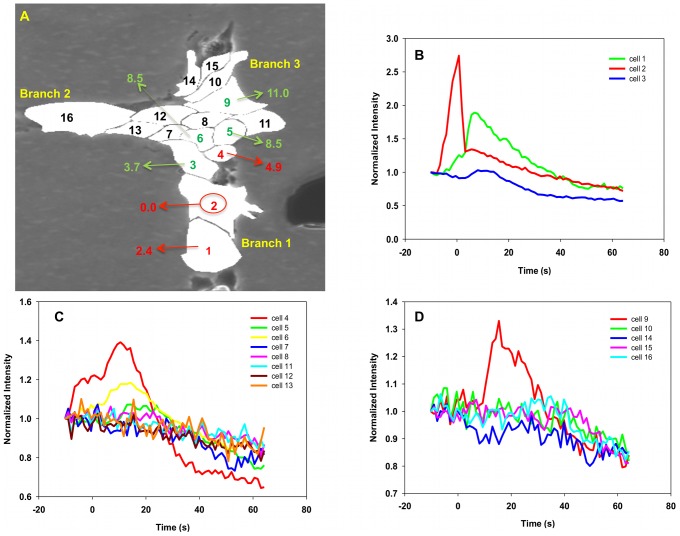
Experiment image 2 and normalized intensity of cells in “T” structure subjected to a single mechano-stimulation. (A) Image of a T structure of cells subjected to single mechano-stimulation. See Figure 8 for detail. (B–D) shows the normalized intensity of fluorescence of representative cells as functions of time. The vertical axis is the dimensionless normalized intensity of fluorescence and the horizontal axis is the time in seconds in intervals of 1.2 s between recordings.

The growth of “T” structures formed by surface-patterning perpendicular single chains of cells does not permit the formation of cellular junctions composed of a single cell. Typically, many cells aggregate at the junction of the three branches forming a cell cluster (see Figure 8 and Figure 9).

#### “T” structure-single stimulation: experimental

In this “T” structure, a single stimulation was applied to one of the branches to determine if the calcium signal can propagate through the junction area and trigger a signal in both of the other branches. We illustrate in Figure 8 and Figure 9 the behavior of “T” structures with two cases. In both cases, the stimulated cell is located in the lower branch (branch 1) at the edge of the junction area. This is done to ensure that there exist paths for the propagation of a calcium pulse to the other branches that do not exceed 4 to 5 cells in length.

In Figure 8 and Video S3, two calcium signals originate from stimulated cell 4 (branch 1) and propagate into two directions, upward and downward. While the downward propagation is similar to that of a single chain although cell 1 (end cell of branch 1) does not respond, probably due to edge effects, and for our purposes, is not relevant. In contrast, a strong pulse propagates upward along a path involving cells 5 to 13. This pulse has similar characteristics as those observed in the non-branched single chains. The calcium-dependent fluorescence lessens in the junction area and does not propagate beyond the junction area into the other two branches. From Figure 8 (D and E), there are two sudden peaks appearing in cell 21 and 26. However, these two peaks are likely individual firing events, which we have observed in several cases, because the neighboring cells do not exhibit significant response and/or the response time is not consistent with the average time of propagation from one cell to the next. A similar behavior occurs in the case of a truncated branch structure as shown in Figure 9. The observed behavior may be understood on the basis of a competition between the intracellular dynamics of CICR and intercellular diffusion that depends on the architecture of the multicellular structure. Indeed, the fluorescence response of a cell results from the release of calcium by ER induced by a cytosolic calcium concentration that has risen above a threshold. This rise is due to an imbalance between inward and outward diffusion fluxes from and to neighboring cells. In a single chain, a cell that is being approached by a calcium wave has only two nearest neighbors and an excess of inward versus outward calcium diffusion will lead to rising cytosolic calcium concentration that will trigger CICR. If many inactivated cells surround a cell of interest such as in a cluster, these cells may serve as calcium sinks drawing calcium away from the cytoplasm of the cell of interest via diffusion. The calcium level in that cell may never reach the threshold needed to induce CICR. In a “T” structure, a calcium pulse propagating along a branch composed of a single chain of cells reaches the junction region composed of a cluster of cells. In this case, the calcium diffusion process transforms from diffusion in a one-dimensional space to diffusion in a two-dimensional space. The larger number of paths for diffusion between cells in the two dimensional region may prevent sufficient accumulation of calcium in any cell to trigger calcium release by ER. At the interface between the one-dimensional and two-dimensional regions and inside the junction cluster, for each cell outward diffusion exceeds inward diffusion and the calcium wave is stopped. These experimental observations provide evidence for an effect of the architecture of multicellular structures on the propagation of calcium waves. We conducted 20 experiments similar to those reported in Figure 8 and Figure 9, and half of them (10/20) showed that the calcium signal could transmit to more than one arm. However, upon detailed characterization of the multicellular structures, it appeared that many of the structures that showed transmission of the calcium signal beyond the junction-cluster region possessed structural imperfections such as stimulated branches that were two-cell wide. It is anticipated that calcium signal propagation from a two-cell wide branch to the junction region could not be considered as transitioning from a one-dimensional to a two-dimensional regions anymore. This will be the focus of a future study.

#### “T” structure-single stimulation: simulation

**Figure 10:**
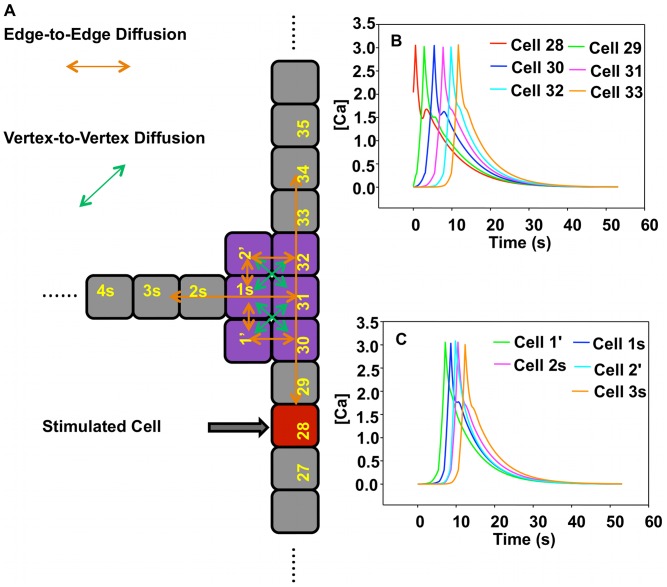
Schematic illustration and simulation results of the model “T” structure subjected to single stimulus with 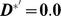. (A) Schematic illustration of the model “T” structure subjected to a single stimulus. Red cell is the stimulated cell. Orange arrows represent the edge-to-edge diffusion. Green arrows represent vertex-to-vertex diffusion. Purple cells highlight the junction cell cluster. (B) Calcium concentration of cells in backbone as a function of time. (C) Calcium concentration of cells 1', 2' and cells in the side branch.

To shed light on the experimentally observed behavior of section “T Structure-Single Stimulus: Experimental” we develop a simplified model that mimics the structural characteristics of the experimental “T” network and can capture the effect of multicellular architecture on the competition between intracellular dynamics and intercellular diffusion. The model architecture is illustrated in Figure 10(A). The structure is composed of a backbone chain containing 61 cells. Cells 30, 31 and 32 are junction cells. A side branch is connected to cell 31 and forms a right angle with the backbone. The side branch contains 30 cells denoted 1 s, 2 s, etc. Two additional cells 1' and 2' are located at the junction of the backbone and the side branch to mimic a cell cluster and multiple junctions. In the model, cells are represented as squares and we differentiate calcium diffusion between cells that are connected by their edges or by their vertices. The diffusion coefficient for edge-to-edge diffusion is the same as that used in modeling a single chain, namely 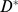. Diffusion through vertices in the junction area, such as for instance between “cell 1 s” in the side branch and cells 30 and 32 in the backbone, is characterized by a dimensionless diffusion coefficient denoted 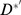. 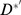 is chosen to be smaller than 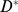 as the diffusion distance along a diagonal direction is larger than the cell-to-cell distance in a chain. Moreover, one also expects the density of gap junctions in a vertex-to-vertex contact area to be lower than in an edge-to-edge area.

**Figure 11:**
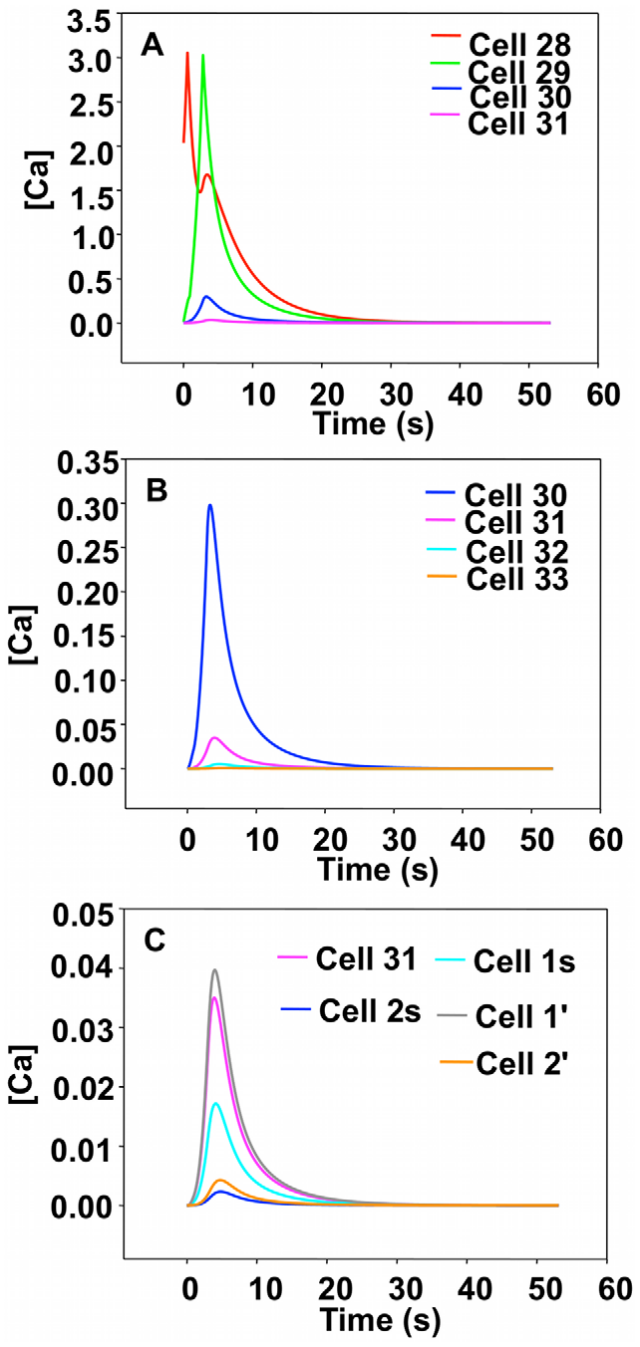
Simulated calcium concentration for cells in the “T” structure subjected to single stimulation with 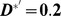. (A) reports the response of cells 28 to 31 in backbone as a function of time. (B) shows the calcium concentration of cells 30 to 33 in backbone as a function of time. Notice the change of scale of the vertical axis. (C) illustrates the calcium concentration of cells 31, 1' and 2' in the junction area and the side branch as a function of time. Again notice the change of scale of the concentration axis.

The reaction-diffusion equations for cells in the junction area are detailed in the Appendix S1. We initially investigate the effect of the value of 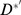 on the behavior of the model system. When 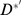 ranges from 0.0 to 0.16, a calcium wave triggered at cell 28 propagates throughout the entire model structure including backbone, side branch and junction cluster (see Figure 10(B) and (C)). For values of 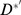 in the range 0.17 to 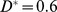, the calcium wave originating at the stimulated cell is unable to propagate beyond the junction region (see Figure 11).

Cells in the junction area and the side branch exhibit very low levels of calcium. Figure 11 indicates for instance that due to the effect of vertex-to-vertex diffusion, the calcium concentration in cell 30 decreases too fast and cannot exceed the threshold *UC_1_* for inducing CICR. Subsequently, the calcium concentration decreases steadily in the junction area. With a larger 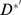 the junction region realizes a conversion from one-dimension-like diffusion to two-dimensional diffusion. The simulated behavior is comparable to that observed experimentally. This suggests that a calcium pulse cannot propagate in a multicellular structure with regions that transition from one-dimensional diffusion (in the lower branch of backbone) to two-dimensional diffusion (in the junction cell cluster). These computational results provide further evidence for an effect of the architecture of multicellular structures on the propagation of calcium waves.

### Double Stimulation of “T” Structure

In section “Single Chain-Dual Stimulation: Experiments” we have demonstrated that two calcium waves cannot cross when propagating toward each other in a chain of endothelial cells. We consider, here, the dual-stimulation of a “T” structure with stimulations located in two separate branches. We address the question of the interaction of the two calcium pulses in the junction area.

#### “T” structure-dual stimulation: experimental

**Figure 12:**
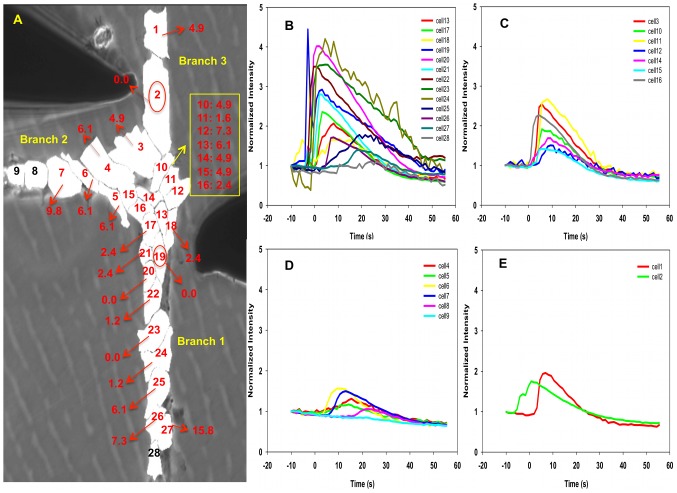
Experiment image and normalized intensity of cells in “T” structure subjected to double stimulation. (A) Image of a T structure of cells subjected to double mechano-stimulation. The red circle identifies the stimulated cells. Red labels indicate the response time individual cells. All the cells exhibited strong fluorescence with response times calculated from the rate of change of the fluorescence intensity. Black labels show cells exhibiting only noise level fluorescence. (B–E) show the normalized intensity of fluorescence of branch 1, cluster area, branch 2 and branch 3, respectively, as functions of time. The vertical axis is the dimensionless normalized intensity of fluorescence and the horizontal axis is the time in seconds in intervals of 1.2 s between recordings.

To facilitate comparison between dual and single stimulation, two separate mechano-stimulations are applied on cells close to the junction in order to ensure that calcium waves would propagate well beyond the junction and into the side branches. Two calcium signals were generated from each stimulated cell. They propagate in two opposite directions. For instance, the calcium signal induced in cell 19 propagates upward toward the junction and downward along the chain of cells that constitute branch 1. Two of the pulses, generated from cell 2 and cell 19 respectively, meet in the junction area. The other pulse terminates at the ends of branches 1 and 3 (see Figure 12).

Figure 12 and Video S4 unambiguously show that nearly every single cell in the “T” structure can support a calcium wave when it is subjected to a dual stimulation. The fluorescence intensity of every cell is strong and possesses the characteristics of pulses observed in the case of the single stimulation of single chains. In contrast to Figure 8 and Figure 9, the cluster area in the junction is not acting as a diffusion sink for the calcium signal and the calcium wave is able to propagate throughout the junction and beyond into branch 2. The signal terminates at cell 8 in branch 2 probably because it has reached its maximum travel distance or due to edge effects. Compared to section “T Structure-Single Stimulus: Experimental”, the dual stimulation launches two calcium pulses toward the junction area. The initial time evolution of the calcium concentration in cells in the junction cluster is now driven by diffusion from several CICR activated cells. The activated cells surrounding a cell of interest in the cluster may serve as calcium sources providing calcium to the cytoplasm of the cell of interest via diffusion. This enables a rise in calcium concentration that can exceed the threshold for triggering subsequent CICR. This rise is now due to an imbalance between outward and inward diffusion fluxes from and to neighboring cells. The two sources of calcium can compensate for the transition from diffusion in the one-dimensional space of the stimulated branches to diffusion in the two-dimensional space of the junction area. We conducted 8 similar experiments and 6 out of 8 (0.75%) showed the same type of behavior.

#### “T” structure-dual stimulation: simulation

**Figure 13:**
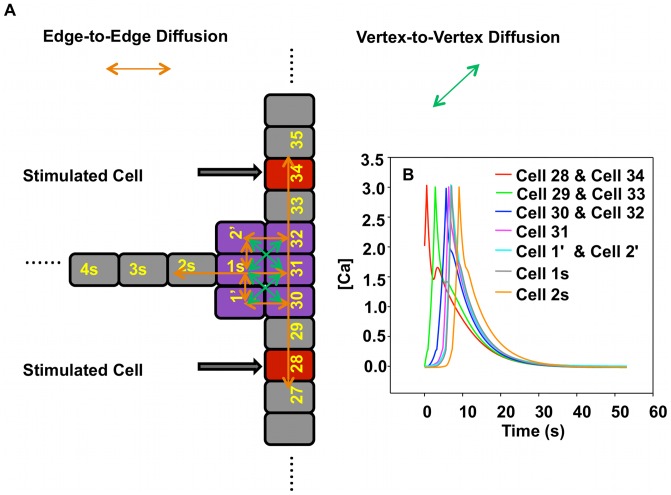
Schematic illustration and simulation results of the model “T” structure subjected to double stimulus. (A) Schematic representation of “T” structure of cells subjected to dual stimulation. Red cells are the stimulated cells. See Figure 10 for additional details. (B) Calcium concentration as a function of time for cells in the vicinity of the junction with 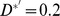.

Figure 13 illustrates the response of the model “T” structure to two stimuli. The model system is identical to that introduced in section “T Structure-Single Stimulus: Simulation”. All cells are initialized to a calcium concentration of 0 but cells 28 and 34, which are given simultaneously an initial concentration of C_0_.

We consider the case of 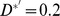 for which we have previously shown the inability to support a propagating calcium wave when it is induced by a single stimulation. Figure 13(B) shows that a calcium wave propagates readily throughout the junction area and into the side branch. Further investigation of the effect of the vertex-to-vertex diffusion coefficient shows that the calcium wave propagation is limited to values of 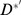 in the range of 0.00 to 0.23. Beyond the value of 0.23, 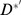 does not allow the “T” structure to support propagation into the side branch as fast diffusion in the cluster would prevent cytosolic calcium concentrations to exceed the CICR threshold. Therefore, in light of the simulations of the “T” structure subjected to single and dual stimulation, agreement with the experimental observations is achieved when 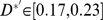. As in the simulations and cell chain experiments, calcium signals propagate through a “T”-like structure of arterioles in the intact microvasculature, a more native architecture, when stimulated chemically by applying acetylcholine as agonist. While our emphasis has been on endothelial cells, this type of behavior likely depends on cell-type, morphology, the nature of the stimulation and environment [23].

## Discussion

In this paper, we study experimentally the propagation of calcium waves in different multicellular structures composed of human umbilical vein endothelial cells (HUVEC). The fabrication of cell-chain based multicellular chain structures relies on organizing multiple cells into specific configurations via selective plasma surface functionalization, which guides cellular attachment. Calcium waves are actuated via mechano-stimulation of selected cells. Calcium wave propagation is characterized by time-resolved fluorescence microscopy. The experimental observations are complemented by modeling and simulation of calcium wave propagation using a diffusion/reaction model. The model of intracellular calcium dynamics is non-linear and mimics the IP_3_-induced calcium release and calcium induced calcium release (CICR). In order to capture the essence of cross-level interactions in calcium signal propagation in multicellular architectures, we only consider a single component model of CICR. This model is different from previous CICR models, which consisted of multiple coupled non-linear differential equations describing the kinetics of IP_3_/Ca^2+^ pumping, release and activation [25], [26]. Nevertheless, the model is capable of capturing most essential features of calcium wave propagation in HUVEC observed in the experiment.

Cell-to-cell interactions are described in this paper via intercellular diffusion through gap junctions. Experimental observation of calcium waves induced by a single mechano-stimulation and propagating along a chain of endothelial cells is used to calibrate the model. Experiments and simulations of chains of cells subjected to dual stimulation (i.e. simultaneous stimulation of two different cells) show that two calcium waves cannot cross each other due to the refractory stage of endothelial cells. The study of more complex multicellular structures utilized “T” structures, which are composed of three side branches joining at a junction. The junction is comprised of cell clusters. In this case, we observe experimentally that when a single cell in one of the side braches is stimulated, the calcium signal does not propagate beyond the junction area. However, when two mechano-stimulations are simultaneously applied on separate branches the calcium signal can propagate through the junction area and beyond well into the third unstimulated side branch of the “T” structure. A computational model of a “T” structure, which includes a cell cluster at the junction, shows the importance of intracellular calcium dynamics and intercellular diffusion in determining the propagation behavior of calcium waves. In particular, the organization of cells in the junction determines the existence of multiple paths for intercellular diffusion, which may affect the accumulation of cytosolic calcium and subsequently the ability of cells to undergo CICR.

In summary, this work demonstrates that the propagation of calcium waves is dependent upon the architecture of multicellular structures. This dependence is due to the competition between intracellular calcium reaction and diffusion, which is affected by the topology through cell connectivity via gap junctions.

## Supporting Information

Appendix S1 **Reaction/Diffusion equations of junction area in T structure.**
(DOCX)

Figure S1 **A capacitive force probe (Nanoscience Inc) was applied for mechanical stimulation of individual HUVECs.** The intensity of fluorescence produced by the stimulated cell shows only very weak correlation with the applied force (The *R square value is determined to be 0.1667 by linear regression analysis*).
(TIF)

Video S1 **Calcium signal propagation in a single chain subjected to single stimulation.**
(AVI)

Video S2 **Calcium signal propagation in a single chain subjected to dual stimulation.**
(AVI)

Video S3 **Calcium signal propagation in T structure subjected to single stimulation.**
(AVI)

Video S4 **Calcium signal propagation in T structure subjected to dual stimulation.**
(AVI)
