# Supplementary material for: Calcium Wave Propagation in Networks of Endothelial Cells: Model-based Theoretical and Experimental Study
Source: PLoS Comput Biol. 2012 Dec 27;8(12):e1002847. doi: 10.1371/journal.pcbi.1002847 (PMC3531288; doi:10.1371/journal.pcbi.1002847)
Supplement: Appendix S1 — Reaction/Diffusion equations of junction area in T structure. (DOCX) [file pcbi.1002847.s001.docx]

# Appendix: Reaction/Diffusion Model of “T” Structure

Here, we detail the discretized reaction-diffusion equations for the “T” structure illustrated in Figures 10(A) and 13 (A). The reaction-diffusion equations are written for the cells in the junction region, namely cells 30 to 32, cell 1s, cells 1’ and 2’. $D^{*}$ and $D^{*'}$ are the dimensionless diffusion coefficient of edge-to-edge diffusion and vertex-to-vertex diffusion, respectively.

The reaction-diffusion equation for cell 30:

$$\begin{matrix} C\left(x_{30},t_{n+1}\right)=C\left(x_{30},t_{n}\right)+D^{*}\left\{\left[C\left(x_{31},t_{n}\right)-2C\left(x_{30},t_{n}\right)+C\left(x_{29},t_{n}\right)\right]+\left[C\left(x_{1^{'}},t_{n}\right)-C\left(x_{30},t_{n}\right)\right]\right\} \\ +D^{*'}\left[C\left(x_{1s},t_{n}\right)-C\left(x_{30},t_{n}\right)\right]-k^{*}\left(C\right)C\left(x_{30},t_{n}\right) \end{matrix}$$

The reaction-diffusion equation for cell 31 is:

$$\begin{matrix} C\left(x_{31},t_{n+1}\right)=C\left(x_{31},t_{n}\right)+D^{*}\left\{\left[C\left(x_{32},t_{n}\right)-2C\left(x_{31},t_{n}\right)+C\left(x_{30},t_{n}\right)\right]+\left[C\left(x_{1s},t_{n}\right)-C\left(x_{31},t_{n}\right)\right]\right\} \\ +D^{*'}\left\{\left[C\left(x_{1^{'}},t_{n}\right)-C\left(x_{31},t_{n}\right)\right]+\left[C\left(x_{2^{'}},t_{n}\right)-C\left(x_{31},t_{n}\right)\right]\right\}-k^{*}\left(C\right)C\left(x_{31},t_{n}\right) \end{matrix}$$

The reaction-diffusion equation for cell 32 is:

$$\begin{matrix} C\left(x_{32},t_{n+1}\right)=C\left(x_{32},t_{n}\right)+D^{*}\left\{\left[C\left(x_{33},t_{n}\right)-2C\left(x_{32},t_{n}\right)+C\left(x_{31},t_{n}\right)\right]+\left[C\left(x_{2^{'}},t_{n}\right)-C\left(x_{32},t_{n}\right)\right]\right\} \\ +D^{*'}\left[C\left(x_{1s},t_{n}\right)-C\left(x_{32},t_{n}\right)\right]-k^{*}\left(C\right)C\left(x_{32},t_{n}\right) \end{matrix}$$

The reaction-diffusion equation for cell 1s in side branch is:

$\begin{matrix} C\left(x_{1s},t_{n+1}\right)=C\left(x_{1s},t_{n}\right)+D^{*}\left\{\left[C\left(x_{2s},t_{n}\right)-2C\left(x_{1s},t_{n}\right)+C\left(x_{31},t_{n}\right)\right]+\left[C\left(x_{2^{'}},t_{n}\right)-2C\left(x_{1s},t_{n}\right)+C\left(x_{1^{'}},t_{n}\right)\right]\right\} \\ +D^{*'}\left\{\left[C\left(x_{32},t_{n}\right)-C\left(x_{1s},t_{n}\right)\right]+\left[C\left(x_{30},t_{n}\right)-C\left(x_{1s},t_{n}\right)\right]\right\}-k^{*}\left(C\right)C\left(x_{1s},t_{n}\right) \end{matrix}$The reaction-diffusion equation for cell 1’ is:

$C\left(x_{1^{'}},t_{n+1}\right)=C\left(x_{1^{'}},t_{n}\right)+D^{*}\left[C\left(x_{1s},t_{n}\right)-2C\left(x_{1^{'}},t_{n}\right)+C\left(x_{30},t_{n}\right)\right]+D^{*'}\left[C\left(x_{31},t_{n}\right)-C\left(x_{1^{'}},t_{n}\right)\right]-k^{*}\left(C\right)C\left(x_{1^{'}},t_{n}\right)$The reaction-diffusion equation for cell 2’ is:

$$C\left(x_{2^{'}},t_{n+1}\right)=C\left(x_{2^{'}},t_{n}\right)+D^{*}\left[C\left(x_{1s},t_{n}\right)-2C\left(x_{2^{'}},t_{n}\right)+C\left(x_{32},t_{n}\right)\right]+D^{*'}\left[C\left(x_{31},t_{n}\right)-C\left(x_{2^{'}},t_{n}\right)\right]-k^{*}\left(C\right)C\left(x_{2^{'}},t_{n}\right)$$
