# Supplementary figures and images for: Calcium Wave Propagation in Networks of Endothelial Cells: Model-based Theoretical and Experimental Study
Source: PLoS Comput Biol. 2012 Dec 27;8(12):e1002847. doi: 10.1371/journal.pcbi.1002847 (PMC3531288; doi:10.1371/journal.pcbi.1002847)

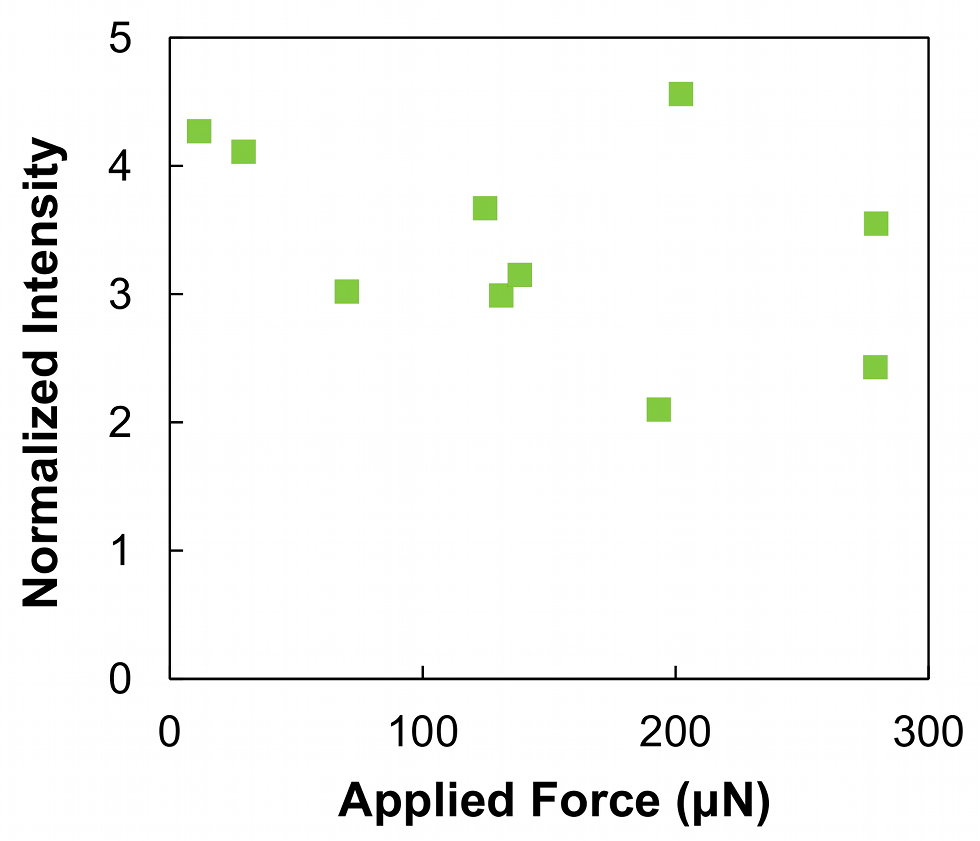

Supplement: Figure S1 — A capacitive force probe (Nanoscience Inc) was applied for mechanical stimulation of individual HUVECs. The intensity of fluorescence produced by the stimulated cell shows only very weak correlation with the applied force (The R square value is determined to be 0.1667 by linear regression analysis). (TIF) [file pcbi.1002847.s002.tif]
